# Two decades of regional trends in vaccination completion and coverage among children aged 12-23 months: an analysis of the Uganda Demographic Health Survey data from 1995 to 2016

**DOI:** 10.1186/s12913-021-07443-8

**Published:** 2022-01-07

**Authors:** Gerald Okello, Jonathan Izudi, Immaculate Ampeire, Frehd Nghania, Carine Dochez, Niel Hens

**Affiliations:** 1 Family Medicine and Population Health, Faculty of Medicine and Health Sciences, University of Antwerp, Antwerp, Belgium; 2 Department of Community Health, Faculty of Medicine, Mbarara University of Science and Technology (MUST), Mbarara, Uganda; 3 Ministry of Health, Kampala, Uganda; 4 Network for Education and Support in Immunisation (NESI), Department of Family Medicine and Population Health, Faculty of Medicine and Health Sciences, University of Antwerp, Antwerp, Belgium; 5 Department of Mathematics and Statistics, Faculty of Sciences, Hasselt University, Hasselt, Belgium

**Keywords:** Vaccination coverage, Vaccination completion, Determinants, Uganda, Regional

## Abstract

**Background:**

Childhood vaccination is an important public health intervention but there is limited information on coverage, trends, and determinants of vaccination completion in Uganda at the regional level. We examined trends in regional vaccination coverage and established the determinants of vaccination completion among children aged 12-23 months in Uganda.

**Methods:**

We analyzed data from the women’s questionnaire for the 1995-2016 Uganda Demographic Health Survey (UDHS). Vaccine completion was defined as having received a dose of Bacillus-Calmette Guerin (BCG) vaccine; three doses of diphtheria, pertussis, and tetanus (DPT) vaccine; three doses of oral polio vaccine (OPV) (excluding OPV given at birth); and one dose of measles vaccine. We performed Chi-square tests to compare vaccination completion by socio-demographic factors stratified by 10 sub-regions: Eastern, East Central, Central 1, Central 2, Kampala, Karamoja, North, Western, West Nile, and Southwest. We performed logistic regression analysis for each of the regions to identify factors associated with vaccination completion at 5% level of statistical significance.

**Results:**

Overall vaccination completion was 48.6% (95%CI, 47.2, 50.1) and ranged from 17.3% in Central 1 to 65.9% in Southwest. Vaccination completion rates declined significantly by 10.4% (95% confidence interval (CI), *− 16.1, − 4.6*) between 1995 and 2000, and increased significantly by 10.0% (95% CI, *4.6, 15.4*) between 2000 and 2006, and by 5.4% (95% CI, *0.2, 10.6*) between 2006 and 2011. Maternal education (secondary or higher level), receipt of tetanus toxoid (TT) during pregnancy, and possession of a child health card were associated with vaccination completion across all the sub-regions. Other factors like place of residence, religious affiliation, household wealth, maternal age, childbirth order, size of child at birth, and place of delivery were associated with vaccination completion but differed between the 10 sub-regions.

**Conclusion:**

Besides considerable regional variations, the vaccination completion rate among children aged 12-23 months in Uganda remains suboptimal despite the availability of vaccines. Maternal education, receipt of TT, and possession of a child health card are associated with a higher likelihood of vaccination completion among children aged 12-23 months in all the regions of Uganda. Interventions to improve the utilization of vaccination services in Uganda should consider these factors.

## Background

Childhood vaccination is a cost-effective public health intervention outlined in the public health armamentarium and a key pillar for achieving Universal Health Coverage (UHC) and the Sustainable Development Goals (SDGs) [1]. Despite interventions to improve vaccination coverage and the subsequent achievements gained in the last 5 years (2008-2014), the third dose coverage of the diphtheria, pertussis, and tetanus (DPT3) vaccine in sub-Saharan Africa (SSA) has plateaued at 72% [2]. For example in 2017, about 10 million children were neither vaccinated or under-vaccinated in the sub-Saharan African region [1] and this potentially exposes them to vaccine-preventable diseases. Consequently, the burden of vaccine-preventable diseases in the region remains high, with an estimated 31 million children under the age of five suffering from vaccine-preventable diseases annually and at least half a million deaths occur due to a lack of access to vaccines [2]. Sub-Saharan Africa, therefore, contributes to between 45 and 50% of severe morbidity and fatalities from leading vaccine-preventable diseases and this estimate is projected to reach 60% in 2030 if no improvements in the deployment of vaccines occur [1].

In Uganda, the Uganda National Expanded Programme for Immunization (UNEPI) established in 1983 is mandated to ensure that all children under the age of 1 year, 10-year-old girls, and all women of childbearing age receive full vaccination of high quality and effective vaccines. Ever since UNEPI has focused on optimal deployment and delivery of vaccines that target childhood preventable diseases namely tuberculosis, polio, whooping cough, diphtheria, tetanus, measles, hepatitis B virus infection, cancer of the cervix, *Haemophilus influenzae* Type B, and pneumococcal infections. To further improve access, coverage, and vaccination completion in Uganda, UNEPI has instituted several vaccination strategies such as community-based outreaches, home-based vaccination, child day plus, and mass immunization campaigns that have been employed over the years. Despite these strategies, vaccination coverage in Uganda has remained relatively low over the past years. For example, DPT-3 and the first dose of measles vaccine coverage have stagnated at 79 and 80%, respectively [3].

National data further show that merely 55% of children aged 12-23 months have received all the basic vaccines. Moreover, vaccination is mandatory for children aged 12-23 months and the expectation is that by the end of 12 months, the children should receive all the vaccine doses. In addition, there are wide regional disparities in the completion of vaccination, with the East Central (Busoga) sub-region standing at 45% and the Karamoja sub-region at 73% [3], leaving the majority of the children either under or not vaccinated. This might increase their risk of morbidity and mortality from vaccine preventable diseases.

Although the determinants of vaccination completion such as maternal education status, antenatal care visits, institutional delivery, maternal age, and household wealth index [4–8] have been well documented, there is limited information on the determinants of vaccination completion at the regional level despite substantial differences in the regional vaccination coverage. Furthermore, previous studies conducted in Uganda [9–12] have focused on factors at the national level while others have looked at specific regions and/or districts besides using data for one period only. Therefore, the studies do not provide a clear picture of the immunization program in the country and have not identified regional variations including the determinants of vaccination completion for targeted action. Furthermore, although vaccination coverage trends have been reported in the Uganda Demographic Health Surveys (UDHS), an analysis of the regional trends in vaccination coverage is limited. Informed by these gaps, we aim to describe the regional trends in vaccination coverage and to identify the determinants of vaccination completion at the regional level among children aged 12-23 months in Uganda using data from Demographic Health Surveys datasets from 1995 to 2016.

## Methods and materials

### Study setting

Uganda is a landlocked country in East Africa, with a total population of 34,634,650 people according to the 2014 census, which is the most recent survey and 45% (15,585,593) of the total population are ≥18 years. There are 134 districts and 6937 health facilities in Uganda, from the latter, 45.2% (3133) are public health facilities. The health facilities are distributed as follows: five national referral hospitals, 14 regional referral hospitals, 169 general hospitals, 194 health center IVs or county-level health facilities, and the rest are health center IIIs or sub-county-level health facilities and health center IIs or parish level health facilities. Uganda has five super-specialized hospitals and two specialized institutes, the Uganda Heart Institute and Uganda Cancer Institute [13]. The country has 10 sub-regions: Central 1, Central 2, East Central, Eastern, Kampala, Karamoja, Northern, Western, West Nile, and Southwest.

### Vaccination schedules in Uganda

According to the UNEPI schedule, a child must receive vaccination 5 times before the first birthday: at birth (BCG, Oral Polio Vaccine (OPV) 0), at 6 weeks (DPT-HEPB-HIB 1, PCV1, Rotavirus vaccine 1, and OPV 1); at 10 weeks (OPV 2, DPT-HEPB-HIB 2, PCV2, Rotavirus vaccine 2); at 14 weeks (OPV 3, DPT-HEPB-HIB 3, IPV, PCV3) and 9 months (measles vaccine). The DPT vaccine is part of a pentavalent vaccine that protects a child from 5 life-threatening diseases namely diphtheria, pertussis, tetanus, hepatitis B, and *Haemophilus Influenzae* type B. Despite the introduction of new vaccines such as the pentavalent DPT-HepB-Hib vaccine, 10-valent pneumococcal conjugate vaccine (PCV), rotavirus vaccine and inactivated polio vaccine (IPV) between 1995 and 2016 to routine vaccination, the schedule for the basic vaccines has remained the same since 1993 [14–18].

### Data source, study design, and population

We conducted a secondary analysis of data from five consecutive UDHS, which are national cross-sectional surveys conducted from 1995 to 2016. The surveys are conducted every 5 years to provide an up-to-date estimate of basic demographic and health indicators. To access the UDHS data, we developed a research concept note and registered the request on the Demographic and Health Surveys (DHS) program website at http://www.DHSprogram.com, stating clearly the research question, the variables needed, time period, country, and the proposed analysis plan. Following the review of the request, we received approval from the DHS Program to retrieve and analyze the dataset. Our study population consisted of 8036 children aged 12-23 months who were alive at the time of the data collection. In the UDHS, a stratified multi-stage sampling technique was applied to select the enumeration areas, both in urban and rural settings and households, using a list of households compiled for all the selected enumeration areas. All women aged 15-49 years in the selected households were eligible to be interviewed using the women’s health questionnaire.

Eligible women were asked questions on background characteristics such as age, level of education, media exposure, birth history, and childhood mortality, knowledge and use of family planning methods, fertility preferences, antenatal care, delivery, and postnatal care, breastfeeding and infant feeding practices, vaccination and childhood illnesses, marriage and sexual activity, women’s work and husband’s background characteristics, awareness and behaviour regarding human immunodeficiency virus (HIV) and Acquired Immunodeficiency Syndrome (AIDS) and other sexually transmitted infections, adult mortality including maternal mortality, and knowledge of tuberculosis and other health issues. In the present study, we only extracted information on background characteristics, and maternal and child health.

### Measurements

The study outcome was vaccination completion, defined as receipt of all of the following antigens: 1) a dose of Bacillus-Calmette Guerin (BCG) vaccine at between birth and 2 weeks; 2) three doses of diphtheria, pertussis, and tetanus (DPT) with the first, second, and third doses at 6, 10, and 14 weeks, respectively; 3) three doses of OPV (excluding OPV given at birth), with the first, second, and third doses at 6, 10, and 14 weeks, respectively; 4) one dose of measles vaccine at 9 months. Any child who has missed any one or more of these vaccines/doses was regarded to had not completed their vaccination schedule.

The independent variables were categorized into four: 1) child related characteristics namely, sex (male or female), birth order (1, 2-3, 4-5, and 6), and size of child at birth (very large, above average, average, below average, and small); 2) maternal related factors such as age in absolute years, education (no education, primary, secondary, and higher), occupation (not working versus working), marital status (never married, married, or separated /divorced/widowed); 3) household related characteristics included wealth index (lowest, second, middle, fourth and highest quantiles), religion (Catholics, Protestants, Muslims, and others), and place of residence (rural versus urban); and 4) the health services-related factors included antenatal care visits (none, 1-3, and ≥ 4), tetanus toxoid injections (none versus yes/ever received), and place of delivery (home, public health facility, private health facility, and others). In the UDHS, data about vaccination coverage were retrieved from the child health card and maternal self-reporting if the child health card was not available.

### Statistical analysis

To determine trends in the regional vaccination coverage, we performed descriptive analyses using the chi-square test to examine regional differences in vaccination coverage, vaccination completion, and the five-year percentage change in vaccination completion rate. To assess the relationship between independent variables and vaccination completion at the regional level, we selected four sub-regions, namely East central, Central 1, Northern, and Southwest, based on their vaccination completion status.

In particular, we included sub-regions with a sample size of at least 50 children aged 12-23 months in a given year. We compared categorical variables with the outcome variable using the Chi-square test and considered variables with probability values (*p*-values) less than 0.05 as statistically significant. All variables that were statistically significant in the bivariate analysis and those that we deemed are biologically plausible to explain the outcome were considered for the multivariate logistic regression modeling. We fitted a stepwise regression model using a backward selection approach and factors with *p*-value < 0.05 were retained in the final model. We further grouped predictors into four categories of household, maternal, child, and health utilization predictors. Crude odds ratio (OR) and adjusted odds ratios (AORs) along with the 95% confidence intervals (CIs) were calculated as a measure of association between the independent variables and vaccination completion. Weighting was performed at every level of analysis to account for the sampling design and the overall analysis was performed in Stata version 14.0 [19] and R version 3.5.1 [20].

## Results

### Trends in vaccination completion across sub-regions and socio-demographic characteristics by year

The proportion of fully vaccinated children aged 12-23 months varied at national and sub-regional levels in Uganda. The proportions ranged from 28.2% (95% CI, 21.0, 36.6) in East-Central sub-region to 64.2% (95% CI, *55.2, 71.9*) in Southwest sub-region in 1995; from 17.3% (95% CI, 10.6, 27.1) in Central 1 sub-region to 47.6% (95% CI, 40.1, 55.3) in Southwest sub-region in 2000; from 38.9% (95%CI, 30.7, 47.9) in East-Central to 52.7% (95%CI, 41.6, 63.4) in Western sub-region in 2006; from 38.6% (95%CI, 29.4, 48.7) in East-Central to 67.5%, (95%CI, 54.4, 78.3) in Kampala in 2011; and from 45.0% (95%CI, 35.8, 54.5) in East-Central to 65.9% (95%CI, 59.1, 72.1) in Southwest in 2016 (Table 1), Karamoja sub-region was excluded due to small sample size. The proportion of fully vaccinated children generally improved in all the sub-regions from 2000 to 2016 after a dramatic decline in most of the sub-regions between 1995 and 2000.

**Table 1:** Vaccination coverage rates of children aged 12-23 months in Uganda by sub-region (1995-2016)

	**Kampala**									
	**1995 (*n*** **= 75)**		**2000 (*n*** **= 68)**		**2006 (*n*** **= 61)**		**2011 (*n*** **= 72)**		**2016 (*n*** **= 126)**	
	**Rate (%)**	***(95%CI)***	**Rate (%)**	***95%CI)***	**Rate (%)**	***95%CI)***	**Rate (%)**	***95%CI)***	**Rate (%)**	***95%CI)***
***Antigens***										
BCG	95.3	*87.7–98.3*	94.1	85.5–97.7	90.1	75.9–96.3	94.8	82.6–98.6	99.2	94.9–99.9
DPT3	78.9	65.0–88.2	58.8	47.0–69.7	68.3	59.1–76.2	76.5	62.3–86.5	79.9	72.1–85.9
OPV3	70.6	60.8–78.8	61.8	50.0–72.3	54.8	45.8–63.5	75.7	66.4–83.1	57.5	47.6–66.9
Measles	78.9	68.1–86.7	70.6	57.6–80.9	69.4	60.3–77.1	83.8	73.5–90.6	81.8	72.4–88.4
All^*^	58.9	*46.1–70.6*	38.2	*26.3–51.8*	45.6	*36.9–54.5*	67.5	*54.4–78.3*	51.4	*41.3–61.3*
	**Central 1**									
	**1995 (*n*** **= 75)**		**2000 (*n*** **= 68)**		**2006 (*n*** **= 61)**		**2011 (*n*** **= 72)**		**2016 (*n*** **= 126)**	
***Antigens***										
BCG	82.6	72.4–89.6	55.6	43.9–66.7	73.9	65.8–80.7	88.4	78.5–94.1	93.3	88.7–96.0
DPT3	70.6	59.3–79.8	23.5	15.9–33.2	51.8	42.7–60.8	70.1	56.7–80.7	74.5	67.8–80.3
OPV3	68.3	58.4–76.7	26.1	17.2–37.3	53.3	44.2–62.1	56	44.6–66.7	63.8	56.5–70.5
Measles	62.5	53.5–70.7	39.7	29.4–50.9	60.9	49.9–70.9	77.8	65.5–86.7	74.9	68.3–80.5
All^*^	55.5	*45.4–65.2*	17.3	*10.6–27.1*	43.2	*33.9–52.9*	46.8	*34.7–59.3*	51.9	*45.3–58.4*
	**Central 2**									
	**1995 (*n*** **= 121)**		**2000 (*n*** **= 102)**		**2006 (*n*** **= 97)**		**2011 (*n*** **= 124)**		**2016 (*n*** **= 268)**	
***Antigens***										
BCG	85.9	78.1–91.3	71.8	62.4–79.6	88.2	78.4–93.9	95.4	89.1–98.1	94.8	89.6–97.5
DPT3	69.3	60.3–77.0	39.9	30.0–50.8	64.8	52.9–75.2	58.6	48.4–68.1	76.4	68.5–82.8
OPV3	67.7	58.2–76.0	43.2	34.6–52.8	61.3	49.1–72.2	53.8	40.7–66.3	57.2	49.0–65.1
Measles	64.6	54.0–73.9	51.7	40.7–62.5	66.3	56.1–75.2	70.9	60.5–79.5	73	63.8–80.6
All^*^	50.4	40.5–60.3	32.3	22.8–43.4	51.1	38.7–63.3	41.7	30.9–53.3	47.1	39.7–54.6
	**East central**									
	**1995 (*n*** **= 130)**		**2000 (*n*** **= 118)**		**2006 (*n*** **= 147)**		**2011 (*n*** **= 135)**		**2016 (*n*** **= 231)**	
***Antigens***										
BCG	75.3	64.7–83.5	79.9	70.2–87.0	88.2	81.7–92.6	96.3	92.3–98.2	97.1	92.3–98.9
DPT3	41.2	33.2–49.8	36.4	23.5–51.6	59.4	48.6–69.3	53.0	44.0–61.7	71.2	63.1–78.1
OPV3	36.8	27.4–47.4	45.6	32.1–59.7	53.2	45.0–61.2	53.1	45.2–60.9	57.0	47.2–66.3
Measles	39.3	31.9–47.2	43.5	31.4–56.4	56.7	48.4–64.6	70.9	63.3–77.4	71.1	62.3–78.6
All^*^	28.2	21.0–36.6	33.0	20.2–48.9	38.9	30.7–47.9	38.6	29.4–48.7	45.0	35.8–54.5
	**Eastern**									
	**1995 (*n*** **= 207)**		**2000 (*n*** **= 214)**		**2006 (*n*** **= 224)**		**2011 (*n*** **= 212)**		**2016 (*n*** **= 476)**	
***Antigens***										
BCG	84.5	77.9–89.4	85.9	79.4–90.6	95.5	91.0–97.8	97.0	92.7–98.8	98.5	97.1–99.2
DPT3	53.9	45.5–62.1	50.8	38.7–62.8	67.2	57.7–75.4	75.4	67.1–82.1	81.7	77.6–85.2
OPV3	53.1	44.9–61.1	65.3	54.6–74.6	63.2	53.4–72.0	61.0	50.4–70.6	66.8	62.3–71.0
Measles	52.7	44.4–60.9	58.5	46.9–69.2	62.6	52.0–72.1	76.6	68.9–82.8	80.9	75.9–85.1
All^*^	37.3	30.0–45.1	41.8	31.4–52.9	46.3	36.1–56.9	52.8	41.3–63.7	56.7	51.3–61.9
	**Northern**									
	**1995 (*n*** **= 138)**		**2000 (*n*** **= 67)**		**2006 (*n*** **= 172)**		**2011 (*n*** **= 115)**		**2016 (*n*** **= 258)**	
***Antigens***										
BCG	81.7	68.1–90.3	69.0	58.8–77.6	96.3	92.6–98.2	94.5	86.6–97.8	97.3	94.7–98.7
DPT3	49.1	28.4–70.1	41.3	28.8–55.0	69.7	59.3–78.3	74.7	63.0–83.6	82.1	76.9–86.3
OPV3	41.6	25.0–60.2	49.7	37.2–62.2	58.9	50.9–66.4	60.4	50.1–69.9	72.9	67.1–78.0
Measles	57.7	40.7–73.1	47.2	32.6–62.2	80.2	71.2–86.9	70.4	56.7–81.2	77.6	71.7–82.5
All^*^	37.0	21.2–56.1	29.1	20.3–39.7	48.5	39.4–57.7	48.3	37.1–59.7	57.5	51.1–63.7
	**Karamoja**									
	**1995 (*n*** **= 9)**		**2000 (*n*** **= 10)**		**2006 (*n*** **= 49)**		**2011 (*n*** **= 54)**		**2016 (*n*** **= 73)**	
***Antigens***										
BCG	58.2	36.8–76.8	100	–	95.9	90.3–98.3	99.9	99.4–100.0	98.8	93.1–99.8
DPT3	25.3	4.2–72.5	81.1	43.7–95.9	65.9	49.0–79.4	92.6	84.0–96.7	87	80.1–91.7
OPV3	25.3	4.2–72.5	95.3	70.7–99.4	64.6	49.0–77.7	67.9	60.2–81.6	79.2	68.6–86.8
Measles	25.3	4.2–72.5	100	–	79.5	69.6–86.8	92.8	84.8–96.7	90.4	82.4–95.0
All^*^	25.3	4.2–72.5	76.4	44.7–92.8	48.8	33.5–64.4	64.5	46.3–79.3	73.5	62.3–82.3
	**West Nile**									
	**1995 (*n*** **= 121)**		**2000 (*n*** **= 103)**		**2006 (*n*** **= 77)**		**2011 (*n*** **= 70)**		**2016 (*n*** **= 178)**	
***Antigens***										
BCG	85.9	75.0–92.5	85.3	75.3–91.7	96.2	91.5–98.3	98.3	93.7–99.6	95.4	90.5–97.9
DPT3	47.2	38.4–56.2	50	39.5–60.5	59.4	48.9–69.1	81.3	71.3–88.4	83.0	76.8–87.8
OPV3	45.6	37.9–53.5	61.9	47.7–74.3	57.1	46.8–66.8	63.0	49.4–74.8	74.8	67.4–80.9
Measles	47.1	39.2–55.1	62.4	55.8–68.6	64.2	53.2–73.8	77.4	68.4–84.5	84.0	77.9–88.7
All^*^	31.9	23.6–41.5	36.3	26.4–47.6	45.3	33.9–57.3	50.7	39.7–61.7	63.7	56.4–70.4
	**Western**									
	**1995 (*n*** **= 183)**		**2000 (*n*** **= 76)**		**2006 (*n*** **= 192)**		**2011 (*n*** **= 157)**		**2016 (*n*** **= 332)**	
***Antigens***										
BCG	84.5	74.1–91.2	78.7	66.6–87.3	91.9	85.4–95.7	94.6	88.2–97.6	95.8	92.7–97.6
DPT3	76	63.5–85.3	50.6	33.3–67.8	71.9	61.0–80.8	77.4	65.4–86.1	76.9	72.5–80.8
OPV3	76.3	63.9–85.3	54.6	38.8–69.5	67.3	57.3–75.9	71.9	58.1–82.6	67.4	62.3–72.1
Measles	68.1	55.7–78.3	72.2	55.8–84.2	75.5	66.0–83.0	80.9	68.4–89.2	85.3	80.8–88.9
All^*^	64	51.4–75.0	39	24.6–55.6	52.7	41.6–63.4	58.8	48.8–68.0	57.5	52.0–62.9
	**Southwest**									
	**1995 (*n*** **= 167)**		**2000 (*n*** **= 199)**		**2006 (*n*** **= 146)**		**2011 (*n*** **= 117)**		**2016 (*n*** **= 262)**	
***Antigens***										
BCG	85.3	77.5–90.7	82	72.0–88.9	86.6	77.8–92.5	87.8	82.3–91.8	97.1	94.2–98.6
DPT3	71.5	61.8–79.5	60.3	51.2–68.7	62.8	51.2–73.1	79.4	71.4–85.6	87.0	81.2–91.1
OPV3	74.2	64.3–82.1	66.2	58.2–73.4	46.7	55.3–73.1	80.3	72.4–86.4	77.1	70.7–82.5
Measles	74.6	65.8–81.8	62.5	55.2–69.2	68.0	57.6–76.8	71.3	60.8–79.9	86.3	80.4–90.4
All^*^	64.2	55.7–71.9	47.6	40.1–55.3	45.7	35.0–56.8	61.4	50.7–71.0	65.9	59.1–72.1

* Completed vaccination: 1 dose of BCG vaccine; 3 doses of DPT vaccine; 3 doses of OPV (excluding OPV at birth); and 1 dose of measles vaccine

**Fig. 1:**
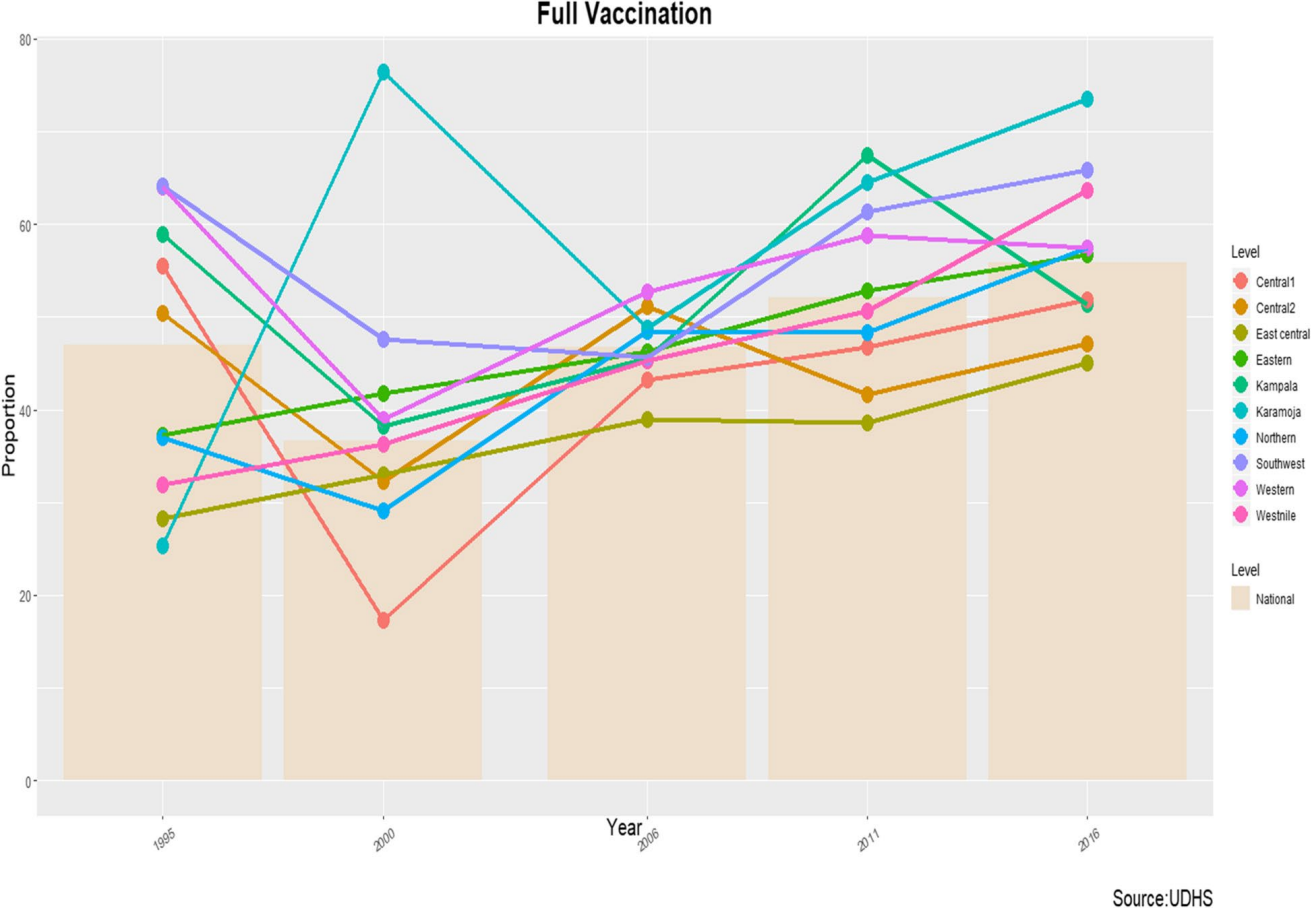
Vaccination Completion Trends among children aged 12-23 months in Uganda by Sub-regions (1995-2016)

The proportion was slightly above the national level in the Western and Southwest sub-regions and increased gradually in Eastern and West Nile sub-regions, and then remained below the national level in East Central, Central 1, and Central 2 sub-regions throughout the period under review (Fig. 1). Overall, the proportion of fully vaccinated children declined significantly from 1995 to 2000 by 10.4% (95%CI, *− 16.1, − 4.6*) and consecutively increased significantly from 2000 to 2006 and 2006-2011 by 10.0% (95%CI, *4.6, 15.4*) and 5.4% (95%CI, *0.2, 10.6*) respectively. By sub-region, the proportion declined significantly in Kampala by 20.7% (95%CI, *− 38.8, − 2.6*), Central 1 by 38.2% (95%CI, − *51.1, − 25.3*), Central 2 by 18.1% (95%CI, − *32.5, − 3.7*), Western by 25.0% (95%CI, − *45.0, − 5.1*) and Southwest by 16.6% (95%CI, *− 27.8, − 5.4*) from 1995 to 2000. However, between 2000 and 2006, the proportion increased significantly in Central 1 by 25.8% (95%CI, *13.2, 38.5*), Central 2 by 18.8% (95%CI, *2.5, 35.1*), and Northern by 19.4% (95%CI, *7.1, 31.5*) and similarly in Kampala by 21.9% (95%CI, *6.9, 36.9*) and Southwest by 15.7%, (95%CI, *0.6, 30.8*) in the period between 2006 and 2011 (Table 2).

**Table 2:** Estimated five-year change (%) in vaccination completion by sub-region and socio-demographic characteristics

	1995–2000	2000–2006	2006-2011	2011-2016
	% change (95%CI)	% change (95%CI)	% change (95%CI)	% change (95%CI)
**Region**				
Kampala	−20.7 (−38, − 2.6)*	+ 7.3 (−8.4, 23.1)	+ 21.9 (6.9, 36.9)*	−16.1 (− 32.4, 0.1)
Central1	− 38.2 (−51.1, − 25.3)*	+ 25.8 (13.2, 38.5)*	+ 3.6 (− 12.2, 19.4)	+ 5.1 (− 8.8, 19.0)
Central2	−18.1 (− 32.5, − 3.7)*	+ 18.8 (2.5, 35.1)*	− 9.4 (−26.3, 7.6)	+ 5.4 (− 8.2, 19.0)
East central	+ 4.8 (− 11.8, 21.5)	+ 5.9 (− 11.1, 23.0)	−0.4 (− 13.4, 12.7)	+ 6.4 (−7.2, 19.9)
Eastern	+ 4.5 (− 8.8, 17.8)	+ 4.6 (− 10.6, 19.8)	+ 6.4 (− 9.0, 21.8)	+ 3.9 (− 8.5, 16.4)
Northern	−7.9 (−28.5, 12.7)	+ 19.4 (6.0, 32.9)*	−0.2 (− 15.0, 14.5)	+ 9.3 (− 3.9, 22.4)
Karamoja	+ 51.1 (4.9, 97.2)*	−27.5 (− 57.2, 2.1)	+ 15.6 (− 7.7, 39.0)	+ 9.0 (− 10.8, 28.8)
West Nile	+ 4.4 (− 9.6, 18.40	+ 9.0 (− 7.0, 25.0)	+ 5.4 (− 10.9, 21.7)	+ 13.0 (− 0.2, 26.2)
Western	− 25.0 (− 45.0, − 5.1)*	+ 13.6 (− 5.7, 33.1)	+ 6.1 (− 8.6, 20.8)	−1.2 (− 12.4, 9.9)
Southwest	− 16.6 (− 27.8, − 5.4)*	−1.9 (− 15.5, 11.5)	+ 15.7 (0.6, 30.8)*	+ 4.5 (− 7.6. 16.7)
**Education**				
None	−8.7 (− 17.3, − 0.1)*	+ 10.8 (2.2, 19.5)*	+ 3.4 (− 7.3, 14.0)	+ 15.0 (4.7, 27.2)*
Primary	− 10.7 (− 18.0, − 3.5)*	+ 9.4 (2.6, 16.1)*	+ 2.3 (− 4.1, 8.7)	+ 4.6 (− 1.0, 10.2)
Secondary	−21.7 (− 34.7, − 8.7)*	+ 8.7 (− 4.3, 21.7)	+ 10.8 (− 0.7, 22.3)	−6.6 (− 16.0, 1.9)
Higher	−25.9 (− 60.1, 8.3)	+ 2.8 (− 24.5, 30.1)	− 0.1 (− 25.1, 25.0)	−1.9 (− 19.6, 15.9)
**Religion**				
Catholic	− 9.9 (− 17.5, − 2.2)*	+ 11.2 (4.1, 18.3)*	+ 9.3 (2.0, 16.5)*	+ 0.6 (− 6.0, 7.1)
Protestants	− 12.8 (− 20.9, − 4.7)*	+ 8.5 (0.3, 16.7)*	+ 5.9 (− 3.0, 14.8)	+ 5.7 (− 2.1, 13.4)
Muslims	−12.4 (− 24.2, − 0.7)*	+ 13.6 (1.1, 26.0)*	+ 8.1 (− 6.0, 22.2)	−1.0 (− 13.4, 11.4)
Others*	+ 8.5 (− 10.6, 27.5)	−0.6 (− 16.7, 16.5)	−8.6 (− 21.7, 4.5)	+ 13.4 (3.5, 23.3)*
**Residence**				
Rural	−9.9 (− 16.2, − 3.6)*	+ 10.0 (4.2, 15.9)*	+ 4.1 (− 1.6, 9.9)	+ 5.8 (0.6, 10.9)*
Urban	−15.0 (− 25.3, − 4.7)*	+ 10.3 (− 0.3, 20.9)	+ 11.7 (0.9, 22.5)*	−7.6 (− 16.5, 12.4)
**Wealth**				
Lowest	+ 7.2 (−4.0, 18.4)	+ 2.1 (− 8.8, 13.0)	+ 6.7 (−3.5, 16.8)	+ 8.2 (− 0.6, 16.9)
Second	−8.3 (− 10.3, 3.7)	+ 7.5 (− 4.1, 19.2)	+ 6.1 (− 3.7, 15.9)	+ 4.1 (− 4.5, 12.6)
Middle	−4.3 (− 15.3, 6.7)	+ 2.8 (− 8.0, 13.7)	+ 1.4 (− 8.8, 11.6)	+ 8.2 (− 1.4, 17.9)
Fourth	−14.5 (− 26.7, − 2.3)*	+ 19.3 (7.1, 31.5)*	+ 3.7 (− 6.5, 14.1)	−0.02 (− 9.2, 9.2)
Highest	−30.5 (− 40.0, − 20.9)*	+ 14.6 (4.9, 24.3)*	+ 9.4 (− 1.3, 20.1)	−2.5 (− 12.2, 7.2)
**Overall**	− 10.4 (− 16.1, − 4.6)*	+ 10.0 (4.6, 15.4)*	+ 5.4 (0.2, 10.6)*	**+ 3.8 (− 0.8, 8.3)**

* Completed vaccination: 1 dose of BCG vaccine; 3 doses of DPT vaccine; 3 doses of OPV (excluding OPV at birth); and 1 dose of measles vaccine

**Fig. 2:**
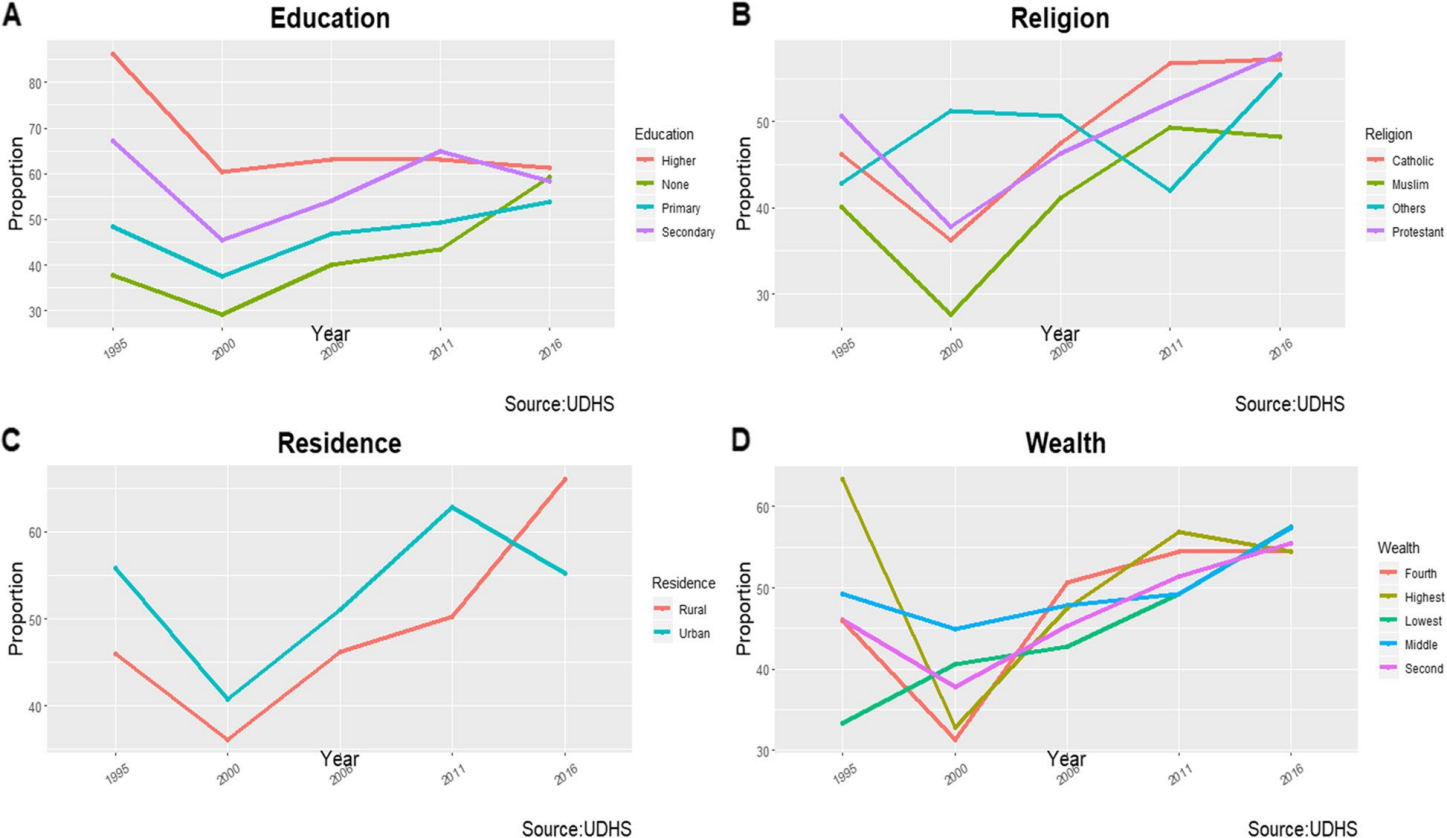
Vaccination completion by socio-demographic characteristics among children aged 12-23 months in Uganda (1995-2016)

The proportion of fully vaccinated children aged 12-23 months was also found to vary by key socio-demographic characteristics. Although the proportion of fully vaccinated children declined significantly across all the four-key socio-demographic characteristics from 1995 to 2000, the proportion generally increased from 2000 to 2016 as shown in Fig. 2. Among those with no education, primary and secondary education, the proportion of fully vaccinated children declined significantly between 1995 and 2000 by 8.7% (95%CI, − *17.3, − 0.1*), 10.7% (95%CI, *− 18.0, − 3.5*) and 21.7% (95%CI, *− 34.7, − 8.7*) respectively, and increased significantly between 2000 and 2006 by 10.8% (95%CI, *2.2, 19.5*) and 9.4% (95%CI, *2.6, 16.1*) among those with no education and primary education respectively and by 15.0% (95%CI, *4.7, 27.2*) among uneducated in the period 2011-2016 (Table 2).

Among Catholics, Protestants and Muslims, the proportion declined significantly by 9.9% (95%CI, − *17.6, − 2.2*), 12.8% (95%CI, − *20.9, − 4.7*) and 12.4% (95%CI, − *24.2, − 0.7*) respectively between 1995 and 2000, and increased significantly in the same groups by 11.2% (95%CI, *4.1, 18.3*), 8.5% (95%CI, *0.3, 16.7*) and 13.6% (95%CI, *1.1, 26.0*) from 2000 to 2006 respectively; however, the proportion only increased significantly among Catholics and other religious groups (Pentecostal, Seventh-day Adventists, Orthodox and Jehovah witness) by 9.3% (95%CI, *2.0, 16.5*) and 13.4% (95%CI, *3.5, 23.3*) between 2006 and 2011 and 2011 and 2016, respectively (Table 2). Among the rural and urban residents, the proportion of fully vaccinated children declined significantly between 1995 and 2000 by 9.9% (95% CI, − *16.2, − 3.6*) and 15.0% (95% CI, − *25.3, − 4.7*) respectively, however, the proportion increased significantly among rural residents by 10.0% (95%CI, *4.2, 15.9*) and 5.8% (95%CI, *0.6, 10.9*) in the period 2000-2006 and 2011-2016 respectively and only increased significantly among the urban residents by 11.7% (95%CI, *0.9, 22.5*) in the period 2006-2011 (Table 2). Finally, the proportion declined significantly between 1995 and 2000 by 14.5% (95% CI, − *26.7, − 2.3*) and 30.5% (95% CI, − *40.0, − 20.9*) among those in the fourth and highest wealth quantile, respectively. During the period 2000-2006, the proportion increased significantly in the same groups by 19.3% (95% CI, *7.1, 31.5*) and 14.6% (95% CI, *4.9, 24.3*), respectively (Table 2).

### Vaccination coverage for basic vaccines (BCG, DPT3, OPV3, and measles)

**Fig. 3:**
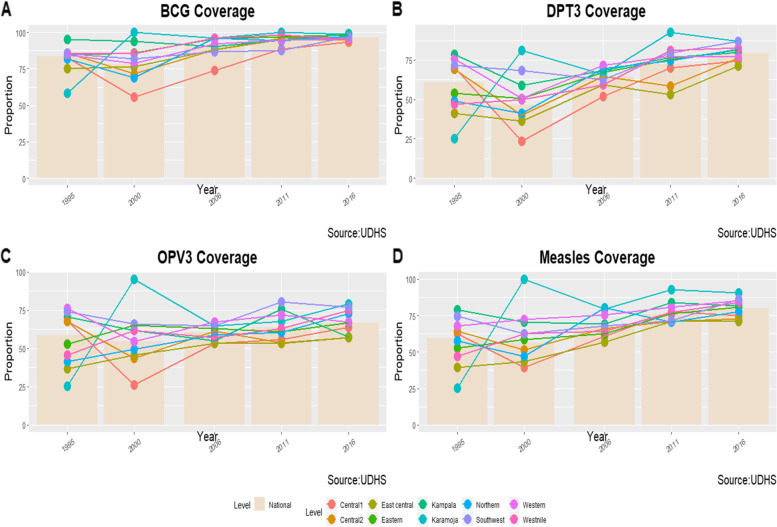
BCG, DPT3, OPV 3, and Measles vaccine Coverage Trends among children aged 12-23 months in Uganda by sub-region (1995-2016)

The vaccination coverage for the basic vaccines (BCG, DPT3, OPV3, and measles) among children aged 12-23 months varied between sub-regions between 1995 and 2016, with the coverage declining in 2000 and then increasing in the subsequent years (Fig. 3). The proportion of children who received BCG ranged from 55.6% (95% CI, *43.9, 66.7*) in Central 1 sub-region in 2000 to 99.2% (95% CI, *94.9, 99.9*) in Kampala sub-region in 2016. The majority of the sub-regions had high BCG coverage of more than 70%, however, some sub-regions had low coverage for instance the Central 1 at 55.6% in 1995 and Northern at 69.0% in 2000 (Table 1).

The proportion of children who received all the three doses of DPT differed between sub-regions ranging from 23.5% (95% CI, 15.9, 33.2) in the Central 1 sub-region in 2000 to 87.0% (95% CI, 81.2, 91.1) in the Southwest sub-region in 2016. However, the coverage of DPT3 was generally lower than 50% in most of the regions in the years 1995 and 2000. For example in East Central (41.2%), West Nile (47.2%) and Northern (49.1%) in 1995 and Central1 (23.5%), East-Central (36.4%), Central 2 (39.9%) and Northern (41.3%) in 2000 (Table 1).

The proportion of children who received all the three doses of OPV excluding OPV0 was also found to vary greatly between regions ranging from 26.1% (95% CI, 17.2, 37.3) in the Central 1 sub-region in 2000 to 80.3% (95%CI, 72.4, 86.4) in Southwest sub-region in 2011. However, most of the sub-regions had low OPV3 coverage of less than 50% in the years 1995 and 2000, for example, East Central (36.8%), Northern (41.6%) and West Nile (45.6%) in 1995, and Central 1 (26.1%), Central 2 (43.2%), East Central (45.6%) and Northern (49.7%) in 2000 (Table 1). Similarly, the proportion of children who received the measles vaccine was found to vary between sub-regions ranging from 39.7% (95% CI, 29.4, 50.9) in the Central 1 sub-region in 2000 to 86.3% (95% CI, 80.4, 90.4) in Southwest sub-region in 2016. However, in the years 1995 and 2000, the measles vaccination coverage was less than 50% in most of the sub-regions, namely East Central (39.3%) and West Nile (47.1%) in 1995 and Central 1 (39.7%), East Central (43.5%), and Northern (47.2%) in 2000 (Table 1). Karamoja sub-region was excluded from the comparison.

### Predictors of vaccination completion

We grouped the predictors of vaccination completion among children aged 12-23 months into four: household, maternal, child, and health services utilization characteristics. At the bivariate analysis, place of residence, maternal level of education, possession of health card, use of tetanus toxoid were statistically significant predictors of vaccination completion in all the four sub-regions. Religious affiliation was borderline while wealth index was a statistically significant predictor of vaccination completion in the Northern sub-region.

Birth order and place of delivery were statistically significant predictors of vaccination completion in Central 1, Northern, and Southwest sub-regions. Lastly, antenatal care visit was a statistically significant predictor in East Central, Northern, and Southwest sub-regions (Table 3).

**Table 3:** Bivariate comparison of predictors of vaccination completion among children aged 12–23 months by sub-region in Uganda (UDHS 1995-2016

Variable	Central 1 (***n*** = 841)			East Central (***n*** = 864)			Northern (***n*** = 831)			Southwest (***n*** = 1008)		
	Children (%)	All*	***p***	Children (%)	All*	***p***	Children (%)	All*	***p***	Children (%)	All*	***p***
***Household Characteristics***												
***Place of Residence***												
Rural	79.6	41.7	0.004*	92.3	36.2	0.012*	92.4	45.7	0.005*	90.8	56.3	0.042*
Urban	20.4	56.5		7.7	51.6		7.6	59.6		9.2	66.7	
***Religious affiliation***												
Catholic	41.1	44.5	0.844	21.2	37.9	0.390	59.9	49.8	0.058*	35.5	54.8	0.196
Protestants	25.8	45.0		42.5	37.5		31.6	39.8		53.1	60.2	
Muslims	21.6	42.2		26.1	33.1		0.4	44.9		2.9	46.8	
Pentecostal + Others	11.5	49.8		10.1	46.7		8.3	52.5		8.5	52.3	
***Wealth index***												
Poorest	4.7	35.6	0.061	24.7	34.8	0.605	46.9	45.2	< 0.001*	12.0	50.0	0.292
Second	10.4	47.5		21.8	34.7		24.6	52.0		19.3	57.1	
Middle	16.3	54.1		21.8	35.6		15.4	30.0		28.3	60.7	
Fourth	27.1	35.3		19.9	41.6		7.6	54.0		27.1	54.4	
Richest	41.6	47.6		11.8	44.1		5.5	73.3		13.4	62.2	
***Maternal Characteristics***												
***Maternal age***												
15–24	41.5	40.7	0.127	39.1	34.1	0.327	39.8	50.6	0.127	30.8	56.2	0.494
25–34	42.1	35.3		44.4	39.8		41.0	46.3		50.6	59.0	
35–49	16.4	47.6		16.5	38.8		19.2	39.7		18.5	54.0	
***Maternal education***												
No education	11.5	33.6	0.001*	16.0	26.6	< 0.001*	17.8	36.5	0.002*	23.1	48.9	0.005*
Primary	58.1	40.8		63.7	34.9		72.1	46.2		61.2	57.8	
Secondary	24.6	55.1		17.6	49.9		8.4	70.0		11.6	71.2	
Higher	5.8	61.9		2.6	78.8		1.7	62.3		4.2	56.4	
***Marital Status***												
Never married	6.3	44.3	0.369	2.9	59.6	0.081	3.1	40.7	0.336	1.8	47.9	0.667
Married	82.1	46.0		89.3	37.2		86.7	47.8		89.7	57.6	
Separated/divorced/widowed	11.6	36.0		7.7	31.1		10.2	39.7		8.5	55.1	
***Maternal working Status***												
Not working	33.8	41.1	0.280	14.3	38.4	0.858	26.2	41.5	0.205	9.6	58.5	0.816
Working	66.2	46.6		85.7	37.2		73.8	48.6		90.4	57.2	
***Child Characteristics***												
***Sex of child***												
Male	56.0	42.5	0.226	51.0	37.1	0.868	47.3	49.2	0.201	50.2	58.7	0.319
Female	44.0	47.6		49.0	37.7		52.7	44.5		49.8	55.5	
***Birth order***												
1	19.5	41.2	0.019*	17.3	38.2	0.987	20.2	51.3	0.032*	15.6	65.9	0.035*
2–3	36.5	53.8		27.3	37.0		27.5	53.0		34.5	54.1	
4–5	23.4	38.2		24.2	36.5		20.5	45.9		21.5	61.2	
6+	20.7	39.5		31.2	38.0		32.0	38.9		28.4	53.3	
***Size at birth***												
Very large	10.1	36.7	0.367	8.0	43.5	0.369	3.1	45.8	0.018*	6.3	60.9	0.001*
Above average	18.9	44.6		27.6	32.1		17.3	57.8		25.8	53.6	
Average	53.2	43.5		43.7	39.8		56.5	44.4		53.6	62.7	
Below average	11.4	52.7		14.2	40.1		18.7	40.3		10.6	41.0	
Very small	6.4	53.9		6.5	30.3		4.4	60.4		3.7	43.7	
***Health Utilization Characteristics***												
***Health card***												
No card	43.1	14.1	< 0.001*	42.9	13.9	< 0.001*	37.6	15.5	< 0.001*	34.8	27.6	< 0.001*
Yes	56.9	68.0		57.1	55.1		62.4	65.5		65.2	73.1	
***Antenatal care Visit***												
None	3.9	32.0	0.364	3.9	15.8	0.022*	3.4	24.7	0.017*	6.5	43.0	0.001*
1–3	41.3	42.9		43.9	35.6		44.2	43.0		49.2	53.1	
4+	54.8	47.0		52.2	40.5		52.2	51.3		44.3	63.9	
***Tetanus Toxoid Injection***												
None	21.6	31.7	0.002*	15.8	22.2	< 0.001*	17.9	29.2	< 0.001*	28.4	47.8	0.001*
Yes	78.4	48.3		84.2	40.2		82.1	50.6		71.6	61.0	
***Place of delivery***												
Home	33.9	35.0	0.008*	31.8	31.6	0.201	49.4	39.3	0.004*	59.1	53.8	0.027*
Public health facilityr	34.3	50.5		45.6	41.3		40.0	55.5		30.2	64.9	
Private health facility	31.3	48.6		21.9	37.6		9.6	48.7		9.4	53.7	
Others	0.5	71.8		0.6	39.3		1.0	47.1		1.2	60.9	

*p < 0.05 at 5% level of statistical significance

After controlling for other variables in the multivariate analysis, the predictors of vaccination completion among children aged 12-23 months varried between the sub-regions. For household characteristics in Central 1, East Central, and Southwest, children aged 12-23 months residing in the urban setting were found to be more likely to complete vaccination compared to those residing in the rural setting AOR (95% CI) 1.85 (1.18-2.89), 1.85 (1.03-3.33) and 1.58 (1.03-2.45). In the Northern sub-region, children aged 12-23 months affiliated to the protestant religion and from middle wealth index households were less likely to complete vaccination compared to those affiliated to the Catholic religion and from the poorest wealth index households AOR (95%CI) 0.67 (0.48-0.93) and 0.52 (0.35-0.79) respectively, while children from highest wealth index households were more likely to complete vaccination compared to those from the lowest wealth index households AOR (95%CI) 3.16 (1.34-7.48) (Table 4). For maternal characteristics, in the Central 1 sub-region, children aged 12-23 months born to mothers aged 25-34 years were more likely to complete vaccination compared to those born to young mothers (15-24 years) AOR (95%CI) 1.51(1.02-2.24). In three sub-regions selected for analysis (Central 1, East Central, and Northern) children aged 12-23 months born to mothers with secondary and higher education were more likely to complete vaccination compared to those born to mothers with no education at all AOR (95%CI) 2.75 (1.47-5.17) and 3.21 (1.43-7.17) in Central 1, 2.94 (1.59-5.45) and 9.50 (2.45-36.85) in East Central, and 4.17(1.69-10.32) and 2.71(1.00-7.32) in Northern, while in Southwest sub-region, only children born to mothers with secondary education were more likely to complete vaccination compared to those born to mothers with no education at all AOR (95%CI) 2.56 (1.51-4.35) (Table 4).

**Table 4:** Multiple logistic regression of predictors of vaccination completion among children aged 12–23 months by sub-regions in Uganda (1995–2016 UDHS)

Variable	Central 1 (***n*** = 841)		East Central (***n*** = 864)		Northern (***n*** = 831)		Southwest (***n*** = 1008)	
	AOR	95%CI	AOR	95%CI	AOR	95%CI	AOR	95%CI
***Household Characteristics***								
***Place of Residence***								
Rural	1.00		1.00		1.00		1.00	
Urban	**1.85**	1.18–2.89**	**1.85**	1.03–3.33**	1.28	0.77–2.12	**1.58**	1.03–2.45**
***Religious affiliation***								
Catholic	1.00		1.00		1.00		1.00	
Protestants	1.02	0.67–1.55	0.97	0.56–1.45	**0.67**	0.48–0.94**	1.27	0.91–1.76
Muslims	0.89	0.55–1.45	0.72	0.42–1.25	0.76	0.10–5.74	0.71	0.36–1.38
Pentecostal*	1.20	0.61–2.37	1.40	0.68–2.85	1.11	0.68–1.79	0.88	0.54–1.45
***Wealth index***								
Poorest	1.00		1.00		1.00		1.00	
Second	1.64	0.54–4.99	1.03	0.54–1.96	1.31	0.94–1.84	1.32	0.77–2.27
Middle	2.13	0.91–4.96	1.08	0.58–2.01	**0.52**	0.35–0.79**	1.55	0.96–2.51
Fourth	0.97	0.38–2.45	1.34	0.76–2.38	1.28	0.58–2.82	1.15	0.69–1.92
Richest	1.34	0.54–3.33	1.32	0.67–2.61	**3.16**	1.34–7.48**	1.44	0.85–2.42
***Maternal Characteristics***								
***Maternal age***								
15-24	1.00		1.00		1.00		1.00	
25-34	**1.51**	1.02–2.24**	1.34	0.94–1.91	0.85	0.59–1.23	1.15	0.83–1.59
35-49	1.22	0.72–2.06	1.53	0.96–2.45	0.66	0.42–1.04	1.02	0.70–1.49
***Maternal education***								
No education	1.00		1.00		1.00		1.00	
Primary	1.45)	0.83–2.55	1.61	0.94–2.75	1.41	0.88–2.25	1.45	0.99–2.11
Secondary	**2.75**	1.47–5.17**	**2.94**	1.59–5.43**	**4.17**	1.69–10.32**	**2.56**	1.51–4.35**
Higher	**3.15**	1.38–7.21**	**9.50**	2.45–36.85**	**2.71**	1.00–7.32**	1.35	0.66–2.76
***Marital Status***								
Never married	1.00		1.00		1.00		1.00	
Married	1.20	0.54–2.65	0.51	0.18–1.46	1.81	0.74–4.44	1.46	0.53–4.01
Separated/widowed	0.82	0.31–2.13	0.37	0.12–1.13	1.12	0.39–3.25	1.33	0.44–4.02
***Child Characteristics***								
***Birth order***								
1	1.00		1.00		1.00		1.00	
2-3	**1.73**	1.04–2.89**	0.93	0.54–1.60	1.09	0.64–1.74	**0.60**	0.40–0.90**
4-5	0.92	0.53–1.58	0.92	0.52–1.64	0.78	0.49–1.27	0.80	0.52–1.25
6+	0.98	0.54–1.76	0.99	0.61–1.60	**0.60**	0.40–0.88**	**0.61**	0.41–0.91**
***Size at birth***								
Very large	1.00		1.00		1.00		1.00	
Above average	1.36	0.70–2.63	0.62	0.30–1.30	1.80	0.71–4.54	0.77	0.42–1.39
Average	1.37	0.77–2.42	0.87	0.43–1.78	1.02	0.40–2.62	1.12	0.61–2.05
Below average	2.01	0.99–4.07	0.87	0.39–1.94	0.84	0.32–2.22	**0.47**	0.23–0.96**
Very small	2.00	0.89–4.52	0.56	0.24–1.32	1.88	0.60–5.86	0.49	0.20–1.24
***Health Utilization Characteristics***								
***Health card***								
No card	1.00		1.00		1.00		1.00	
Yes	**13.14**	8.51–20.2**	**7.30**	4.96–10.75**	**10.56**	6.18–18.06**	**6.93**	4.93–9.73**
***Antenatal care Visit***								
None	1.00		1.00		1.00		1.00	
1-3	0.85	0.34–2.15	1.54	0.59–4.00	0.84	0.22–3.26	0.97	0.51–1.85
4+	0.91	0.35–2.34	1.63	0.61–4.32	1.15	0.29–4.52	1.44	0.73–2.83
***Tetanus Toxoid Injection***								
None	1.00		1.00		1.00		1.00	
Yes	**1.87**	1.08–3.26**	**1.91**	1.14–3.22**	**2.63**	1.56–4.42**	**1.48**	1.01–2.17**
***Place of delivery***								
Home	1.00		1.00		1.00		1.00	
Public health facility	1.57	0.95–2.6	1.32	0.76–2.30	1.22	0.79–1.87	1.10	0.77–1.59
Private health facility	**1.71**	1.05–2.78**	1.23	0.77–1.98	0.86	0.51–1.45	0.68	0.40–1.14
Others	**6.38**	1.28–31.87**	1.92	0.55–6.64	1.50	0.43–5.25	1.11	0.46–2.67

*p < 0.05; ** *p* < 0.01 at 5% level of statical significance

Child characteristics were found to influence vaccination completion differently in the sub-regions. In the Central 1 sub-region, children in the 2-3 birth order were more likely to complete vaccination AOR (95%CI) 1.73 (1.04-2.89) and less likely to complete in Southwest sub-region AOR (95%CI) 0.60 (0.40-0.90) compared to those in the 1st birth order, while in the Northern and Southwest sub-regions, children in the 6+ birth order were less likely to complete vaccination AOR (95%CI) 0.60 (0.41-0.88) and 0.61 (0.41-0.91) respectively compared those in the 1st birth order. We also found that in the Southwest sub-region, children with a birth weight below average (2.5 kg) were less likely to complete vaccination AOR (95%CI) 0.47 (0.23-0.96) compared to those with very large birth weight (Table 4). Finally, the health utilization characteristics were also found to influence vaccination completion differently in the sub-regions. In all four sub-regions children who had a child health card were more likely to complete vaccination AOR (95CI) 13.14 (8.51-20.26) in Central 1, 7.30 (4.96-10.75) in East Central, 10.56 (6.18-18.06) in Northern, and 6.93 (4.93-9.73) in Southwest compared to children who did not have the health card. Similarly, children whose mothers received a tetanus toxoid injection during the pregnancy were more likely to complete vaccination across the four sub-regions AOR (95%CI) 1.87 (1.08-3.26) in Central 1, 1.91 (1.14-3.22) in East Central, 2.63 (1.56-4.42) in Northern and 1.48 (1.01-2.17) in Southwest compared to those whose mothers did not receive the tetanus toxoid injection during pregnancy. Antenatal care visits did not have a statistically significant influence on vaccination completion across the four regions, similarly, place of delivery did not have a statistically significant influence on vaccination completion in three sub-regions (East Central, Northern, and Southwest). However, in Central 1, children delivered at private health facilities and others (roadside) were more likely to complete vaccination for AOR (95%CI) 1.71 (1.05-2.78) and 6.38 (1.28-31.87) respectively compared to those born at home (Table 4).

## Discussion

Our study examined regional trends in vaccination coverage and identified the determinants of vaccination completion at the regional level among children aged 12-23 months using five Demographic Health Surveys in Uganda from 1995 to 2016. We found substantial regional disparities in vaccination completion and vaccination coverage of basic vaccines. We found that the vaccination completion and vaccination coverage rates declined between 1995 and 2000 across the majority of the sub-regions, but with notable improvements across almost all the sub-region between 2000 and 2016, except for Kampala and Western sub-regions where we observed a decline between 2011 and 2016, and between 2006 and 2011 for Central 2 region.

Our data show that vaccination completion and vaccination coverage rates are slightly above the national rates in Western and Southwest sub-regions, although we observed lower rates in East Central, Central 1, and Central 2. The rest of the sub-regions had rates within the range of the national rates. The dramatic decline in vaccination completion and vaccination coverage between 1995 and 2000 could be attributed to a disruption in the healthcare system which was characterized by the adoption of a decentralization approach of health system delivery, poor injection safety measures, and limited funding according to an adhoc report. For instance during the decentralization process in Uganda, the focus of the Uganda Ministry of Health shifted from preventive and primary health care services to private curative services. Furthermore, the decentralization process resulted in reduced funding to services traditionally addressed by vertical programs, an example was immunization program hence reduced number of vaccinators to deliver routine and outreach immunization services [21]. The notable progress in vaccination completion and coverage was a result of the revitalization plan of Uganda expanded immunization program which started around 1999 when there was the remarkable strengthening of infrastructure for delivery of vaccination services, rehabilitation of the cold chain and communication services, capacity building, improving injection safety practices, introducing supplementary immunization activities, renewing high-level advocacy, improving surveillance and adding more vaccines [22].

Besides, investments into the program from GAVI or the Vaccine Alliance, sustainable outreach services, and the implementation of reaching every district (RED) approach [23] also contributed to improvements in vaccination completion and coverage between 2000 and 2016.

Furthermore, vaccination completion declined between 1995 and 2000 by key socio-demographic characteristics, namely education, residence, religious affiliation, and wealth index and increase from 2000 to 2016. By 2016, there were almost no disparities in vaccination completion rates by education level, wealth index, and religious affiliation. This could also be due to the above-mentioned reasons; however, a striking result was noted, the vaccination completion rate declined in the urban setting below the rural setting between 2011 and 2016. This decline could be attributed to much more focus on increasing access to health services in the rural settings by constructing more health facilities and implementation of sustainable outreach services in the rural settings.

The study also revealed variations in the predictors of vaccination completion in the four selected sub-regions. We found the maternal level of education, possession of child health card, and receipt of TT injection during pregnancy are associated with vaccination completion in all four sub-regions. Maternal education implies that the mother is able to comprehend information received, which in turn may lead to increased awareness, change of attitude, autonomy and control and decision making. Possession of the child health card enables the parents to remember the child’s immunization schedule, therefore the card acts as a reminder to parents. Mothers who received TT injection during pregnancy are aware of the benefits of immunization and therefore will ensure that their children complete their immunization. These findings are consistent with studies conducted in Togo, Ethiopia, disaster-affected communities in Pakistan, Malawi, and Zimbabwe [24–28]. The findings are also consistent with a study conducted in a Somali national regional state in Ethiopia that found receiving TT injection predicts vaccination completion [29]. The other predictors of vaccination completion were the place of residence in the three sub-regions of Central 1, East, central, and Southwest where children from urban settings were more likely to complete vaccination.

This may be attributed to the higher concentration of both public and private health facilities in the three sub-regions, with the private facilities providing free immunization services thus reducing the distance to the immunization centers. This finding is consistent with a study conducted in Ethiopia [29] but differs from a study in Bolivia that found children from urban settings were less likely to complete vaccination [30]. The childbirth order was a predictor in the three sub-regions of Central 1, Northern, and Southwest and this is consistent with a study conducted in Zimbabwe [28]. Maternal age and place of delivery were only statistically significant in the Central1 sub-region. This finding is similar to the findings from studies conducted in East Africa [11, 28, 29]. Elsewhere in Greece, a study also found that older mothers tended to complete vaccination compared to younger mothers [31]. Our data show that a child coming from a household affiliated to the Protestant religion and a household with middle and richest wealth status is more likely to complete vaccination in the northern sub-region only. This finding is consistent with studies conducted in Togo, Pakistan, Malawi, and Zimbabwe [24, 26–28]. Interestingly, in our study, antenatal care visits are not associated with vaccination completion contrary to studies conducted in Ethiopia [25], Eastern Uganda [28], and Zimbabwe [32].

### Study strengths and limitations

This study has several strengths and limitations. By combining datasets from five demographic health surveys, the study had a large sample size to produce unbiased estimates and trend analyses. The data were collected using a standardized data collection procedure and pre-tested tool with good psychometric properties which has been validated at the international level for adoption at the national level. However, there are limitations. We noted that for certain sub-regions such as the Karamoja region, the sample size was too small to detect a statistically significant difference. We, therefore, excluded data from the region in the descriptive analysis of vaccination completion and coverage to minimize biased results. The data analyzed was from cross-sectional surveys so our findings lack temporal association in that they cannot demonstrate a cause-effect relationship. The possibility of reporting biases during the primary survey and its effects on the current study is another important limitation to consider.

We did not have qualitative data to support or explain the findings and this should be a subject for further research.

## Conclusion

This study shows that the vaccination completion rate among children aged 12-23 months in Uganda has slightly improved across all the sub-regions. Although vaccination services are readily available, the vaccination completion rate is suboptimal and differs across the majority of the sub-regions. Maternal level of education, possession of a child health card, and receipt of TT injection during pregnancy are associated with vaccination completion among children 12-23 months across all the sub-regions.. Other factors like place of residence, religious affiliation, household wealth, maternal age, childbirth order, size of child at birth, and place of delivery were associated with vaccination completion but differed between the 10 sub-regions. We concluded that there is a need for the Ministry of Health and other implementing agencies to implement targeted interventions to improve the vaccination completion rates in Uganda. Besides, there is a need to conduct additional studies to explore reasons at individual, community, and health care levels that account for failure to complete vaccination despite the availability of vaccination services.

## Data Availability

The datasets generated and/or analysed during the current study are available in the www.dhsprogram.com repository.

## Abbreviations

AOR: Adjusted odds ratio
BCG: Bacillus-Guerine Calmette
CI: Confidence interval
DPT: Diptheria, Purtusis, and Tetanus
HEPB: Hepatitis B
IPV: Inactivated Polio Vacine
OPV: Oral Polio Vaccine
OR: Odds Ratio
PCV: Pneunonia Conugate Vaccine
TT: Tetanus toxoid
UHC: Universal Health Coverage
UNEPI: Uganda National Expanded Program for Immunization
SDGs: Sustainable Development Goals
UDHS: Uganda Demographic Health Surveys
